# Anti-CD20 Disrupts Meningeal B-Cell Aggregates in a Model of Secondary Progressive Multiple Sclerosis

**DOI:** 10.1212/NXI.0000000000000975

**Published:** 2021-03-02

**Authors:** Jay Roodselaar, Yifan Zhou, David Leppert, Anja E. Hauser, Eduard Urich, Daniel C. Anthony

**Affiliations:** From the Department of Pharmacology (J.R., Y.Z.), University of Oxford; University of Basel (D.L.), Switzerland; Deutsches Rheumaforschungszentrum (DRFZ) and Department of Rheumatology and Clinical Immunology, Charité Universitätsmedizin Berlin (J.R., A.E.H.), Germany; Roche Innovation Center (E.U.), Basel, Switzerland; and Department of Pharmacology (D.C.A.), University of Oxford, UK.

## Abstract

**Objective:**

Therapies targeting B cells have been used in the clinic for multiple sclerosis (MS). In patients with relapsing MS, anti-CD20 therapy often suppresses relapse activity; yet, their effect on disease progression has been disappointing. Most anti-CD20 therapeutic antibodies are type I, but within the unique microenvironment of the brain, type II antibodies may be more beneficial, as type II antibodies exhibit reduced complement-dependent cytotoxicity and they have an increased capacity to induce direct cell death that is independent of the host immune response.

**Methods:**

We compared the effect of type I with type II anti-CD20 therapy in a new rodent model of secondary progressive MS (SPMS), which recapitulates the principal histopathologic features of MS including meningeal B-cell aggregates. Focal MS-like lesions were induced by injecting heat-killed *Mycobacterium tuberculosis* into the piriform cortex of MOG-immunized mice. Groups of mice were treated with anti-CD20 antibodies (type I [rituxumab, 10 mg/kg] or type II [GA101, 10 mg/kg]) 4 weeks after lesion initiation, and outcomes were evaluated by immunohistochemistry.

**Results:**

Anti-CD20 therapy decreased the extent of glial activation, significantly decreased the number of B and T lymphocytes in the lesion, and resulted in disruption of the meningeal aggregates. Moreover, at the given dose, the type II anti-CD20 therapy was more efficacious than the type I and also protected against neuronal death.

**Conclusions:**

These results indicate that anti-CD20 may be an effective therapy for SPMS with B-cell aggregates and that the elimination of CD20^+^ B cells alone is sufficient to cause disruption of aggregates in the brain.

Anti-CD20 therapy reduces relapses in relapsing MS (RMS), but in secondary progressive MS (SPMS), the efficacy of anti-CD20 is less certain.^1–3^ It also remains unclear what impact anti-CD20 has on the histopathologic features of SPMS, such as meningeal tertiary lymphoid–like structures (TLSs). TLSs are associated with more aggressive disease progression, increased demyelination, neuronal damage, and microglial activation.^4–8^ The B cells in meningeal aggregates in SPMS are thought to contribute to disease pathology by the production of inflammatory mediators and provide a hub for antigen presentation.^9–11^

Our knowledge of the formation and evolution of TLS is based on postmortem studies, and in vivo detection of TLS in patients remains challenging. Here, we report the development of a new focal model that recapitulates the histologic features of SPMS with TLS, in which we examined the efficacy of anti-CD20 therapy. We compared the action of the type I antibody (Ab) rituximab (RTX) with the type II Ab GA101 on the development of SPMS-like lesions. Compared with type I anti-CD20 antibodies, type II antibodies exhibit reduced complement-dependent cytotoxicity (CDC) and have a direct cell killing property while still binding to the same epitope on the CD20 molecule.^12–14^ To date, no direct comparison between the efficacy of type I and type II has been investigated in MS or in any of its models.^13,15,16^ Thus, we compared the action of a clinically relevant dose of a type I anti-CD20 Ab with a type II Ab on the development of SPMS-like lesions.

## Methods

### Induction of the DTH-TLS Model

Adult humanized (hu)CD20 C57Bl/6, expressing human CD20 exclusively on B cells, and their wildtype littermates, ±25 g (Janvier Labs, France), were used.^17^ Delayed-type hypersensitivity (DTH)-TLS lesions were established as follows: on day -12, animals were immunized with an emulsion of MOG_35-55_peptide (SP-5360-5, Innovagen, Sweden) (0.5 mg/mL in saline) and CFA with heat-killed *Mycobacterium tuberculosis* (TB) (Difco H37Ra, Becton Dickinson (BD), UK) (5 mg/mL in the total volume) intradermally in the hind legs. On day 0, mice received a stereotaxic injection of heat-killed TB (8.8 mg/mL in saline) in the piriform cortex (Bregma: AP +0.7, ML +2.7, DV −3.5). Briefly, anesthesia was induced and maintained at 2%–2.5% isoflurane in oxygen. The head of the animal was shaved, sterilized, and placed in a stereotaxic frame under an operating microscope, and bupivacaine was administered locally. A dental drill was used to make a burr hole in the skull. A finely pulled glass microcapillary was used to inject 1 μL of substance, which was delivered over a period of approximately 5 minutes, before the wound was sutured. Cages were checked daily to ensure animal welfare by evaluating body weight and physical well-being.

### Treatment With Anti-CD20

Treatment started 40 days after lesion induction. Animals were randomly assigned to be treated with either type II anti-CD20 (GA101 [10 mg/kg, n = 11]), type I anti-CD20 (RTX [10 mg/kg, n = 8]), or vehicle (saline) (n = 13). Patients with demyelinating disease are often treated with anti-CD20 therapy at a similar dose per kg (1,000 mg IV). Animals received 3 IV injections (100 μL) via the tail vein spread over 15 days. Animals were perfused 18 days after initiation of treatment.

### Tissue Sample Collection and Preparation

Animals were deeply anesthetized with pentobarbital. Blood was collected via cardiac puncture and transferred to EDTA-coated tubes. Animals were then transcardially perfused with heparinized saline, followed by 4% paraformaldehyde (PFA). Organs postfixed in 4% PFA at 4°C and dehydrated in 30% sucrose afterward. Organs were embedded in OCT and stored at −20°C.

### Blood Analysis

Blood analysis was performed using an ABX Pentra 60 hemocytometer (Horiba, Japan). The average of 3 readings was calculated for the number of total circulating leukocytes and lymphocytes.

### Histology and Analysis

Ten-micrometer-thick cryosections were cut (Leica Microsystems, UK), and immunohistochemistry was performed using a streptavidin-biotin-peroxidase method,^18^ using the following primary antibodies: microglia/macrophages, Iba-1, 1:2,000 Abcam; astrocytes, glial fibrillary acidic protein 1:800 Dako; B cells, B220 1:800 BD; T cells, CD3 1:200 BD; myelin, MBP 1:1,000 Abcam; neurons, NeuN 1:1,000 Abcam; proliferation, ki67 1:200 Merck; plasma cells, CD138-PE 1:50 Miltenyi; and activated macrophages, Mac-2 1:100 BioLegend. Sections were counterstained with cresyl violet, dehydrated, cleared, and mounted with Dibutylphthalate Polystyrene Xylene (DPX) mounting medium. For immunofluorescence, slides were hydrated, blocked in serum, and incubated with primary antibodies. Fluorochrome-conjugated secondary antibodies were used for signal detection. Slides were mounted with Vectashield mounting medium with 4',6-diamidino-2-phenylindole (DAPI).

### Flow Cytometry

Half the spleen was isolated and stored in Roswell Park Memorial Institute 1640 medium containing 1% FBS on ice. A 70-μm cell strainer was used to obtain a single-cell suspension. Red blood cells were lysed, and splenocytes were blocked with Fc block (BD sciences) and stained with anti-CD19-Alexa Fluor 488. All flow cytometric data were collected using a FACSCanto II (BD Biosciences) and analyzed using FlowJo software (v.10.3.0, Tree Star, Inc).

### Statistical Analysis

All data were analyzed with the GraphPad Prism software (v.6, GraphPad Software Inc). To identify differences between the groups, an unpaired *t* test or 1-way analysis of variance with Tukey multiple comparison test was used. All data are presented as mean ± standard error of the mean and were considered statistically significant if *p* < 0.05.

### Standard Protocol Approvals, Registrations, and Patient Consents

Mice were bred and maintained at the University of Oxford's Biomedical Services Department*.* All animal procedures were performed with ethical approval under UK HO Licence P996B4A4E.

### Data Availability

Data not shown will be shared on reasonable request.

## Results

### Chronically Activated Microglia and Astrocytes Spread Out Over Time

**Figure 1:**
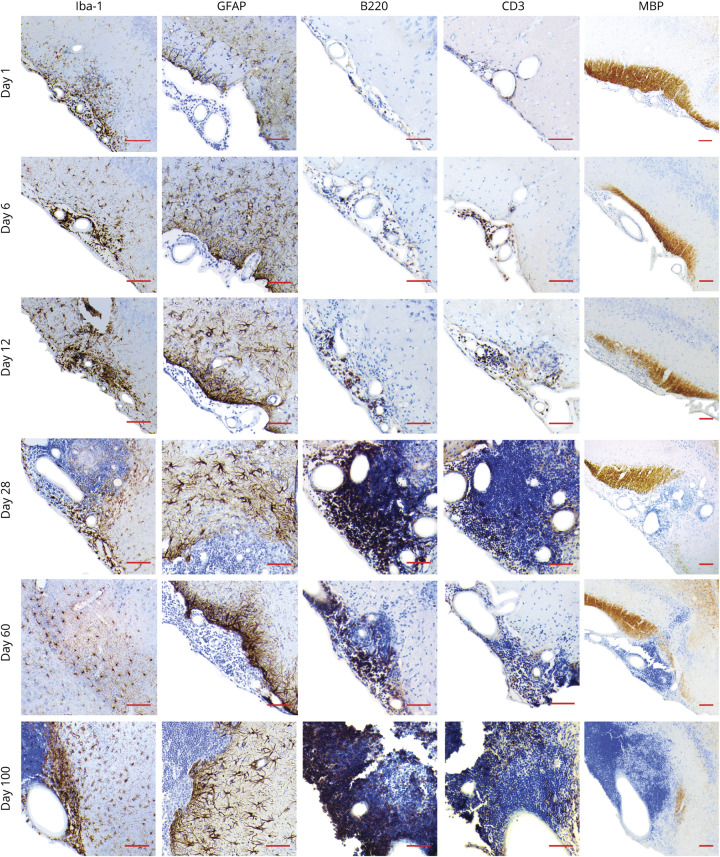
DTH-TLS Model Development Characterization Focal MS-like brain lesions with TLS-like structures were established in mice. Mice were perfused on days 1, 6, 12, 28, 60, and 100, after which brains were analyzed for microglia (Iba-1), astrocytes (glial fibrillary acidic protein), B cells (B220), T cells (CD3), and myelin (MBP) by immunohistochemistry. The DTH-TLS model features a gradually expanding area of activated microglia and astrocytes, an increasingly growing lymphocyte compartment with B- and T-cell compartmentalization, and increasingly worsening demyelination. Representative micrographs are shown of n = 4 per time point. Red scale bars represent 100 μm. TLS = tertiary lymphoid structures.

As in MS, the DTH-TLS model displayed chronic, slowly expanding, microglial activation (figure 1). From day 1 onward, microglia adopted an activated phenotype, characterized by their amoeboid morphology and the retraction and thickening of their processes. The reactive morphology appeared with clustering and nodule formation in later time points. Microglial activation was the most intense around the lesion cores and around the meningeal lymphocyte aggregates.

Astrocytes followed the microglial activation pattern (figure 1). They adopted a reactive phenotype, characterized by their hypertrophied cell body and thickened processes. The area covered by astrocytes increased over time and tended to spread out even further than the area covered by microglia. Astrocyte density was highest around the meningeal aggregates and around inflamed blood vessels, especially in the later time points. At these stages, the formation of glial scars was also evident, recognized by the elongated shape of the astrocytes, resembling a barrier between the lymphocytic aggregates and the brain parenchyma.

### B and T Cells Increase in Number Over Time and Form TLS-Like Structures in the Meninges in the DTH-TLS Model

Anti-B220 and anti-CD3 were used to identify B and T cells within the aggregates. Both B and T cells were present in the meninges even 1 day after lesion induction, and their numbers increased over time (figure 1). B and T cells were not exclusively found in the meninges. In the brain parenchyma, B and T cells were mostly present in the cuffs of inflamed blood vessels. Smaller cell clusters were found throughout the parenchyma. In the meningeal aggregates, the proportion of B and T cells appeared similar in the earlier time points, but as time progressed, the proportion of B cells increased. Concurrently, the pattern of the aggregates changed—from being scattered randomly, B and T cells started to become compartmentalized from each other, which was especially evident from d60. As time progressed, no shrinkage of the aggregates or reduction in the number of lymphocytes was observed.

### Worsening Demyelination in the Olfactory Tract of Mice With the DTH-TLS Model

Anti-MBP was used to visualize myelin in the brains of mice with DTH-TLS lesions. With the spread of inflammation, demyelination in the lateral olfactory tract became more prominent over time (figure 1). From day 12 onward, demyelination was clearly visible and was associated with an increased number of cells in the cortex and the meninges surrounding the olfactory tract. Demyelination kept worsening, and at day 100, very little MBP remained. Myelin loss was associated with meningeal TLS growth.

### Proliferating B Cells Were Present in Meningeal TLS in the DTH-TLS Model

**Figure 2:**
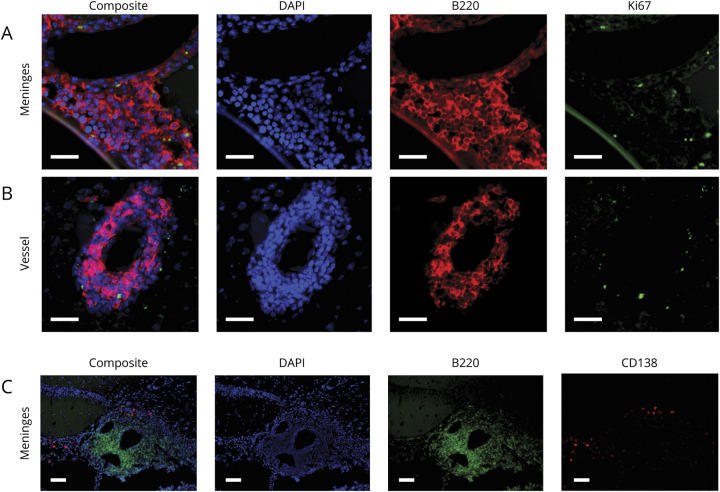
Plasma Cells and Proliferating B Cells Can Be Observed in DTH-TLS Lesions Focal MS-like DTH-TLS lesions were established in mice. Brains were stained for B cells (B220 in red), proliferation (Ki67 in green), and DAPI (blue) using immunofluorescence. The DTH-TLS model features proliferating B cells in both the meninges (A) and in vessel cuffs (B). CD138+ plasma cells were also present in the lesions (in red), outside of the area occupied by B cells (in green) (C). White scale bars represent 100 μm. TLS = tertiary lymphoid structures.

Next, brain sections were double labeled for B cells (anti-B220, red) and proliferation (anti-Ki67, green) using immunofluorescence. Ki67^+^B220^+^ cells were identified in the meningeal aggregates and in cuffs around blood vessels (figure 2, A and B). The Ki67 stain colocalized with the DAPI-stained cell nuclei. Furthermore, double labeling for B cells and plasma cells (anti-CD138) showed that plasma cells were present in the meningeal aggregates, outside of the B-cell zone, like in lymphoid organs (figure 2C and e-1, links.lww.com/NXI/A431).

### No Blood-Brain Barrier Breakdown Detected With Gd-Enhanced MRI in the DTH-TLS Model on d100

To assess the state of the blood-brain barrier (BBB) in the most clinically relevant way, Gd-enhanced MRI was performed. At day 100, postcontrast T1-weighted images revealed no gadolinium enhancement in the lesion area in any of the animals, indicating no BBB breakdown. Some enhancement could be seen in the simple difference between the pregadolinium and postgadolinium images, but when corrected for movement, these changes were no longer present (figure e-1, links.lww.com/NXI/A431).

### Anti-CD20 Depletes Peripheral B Cells

**Figure 3:**
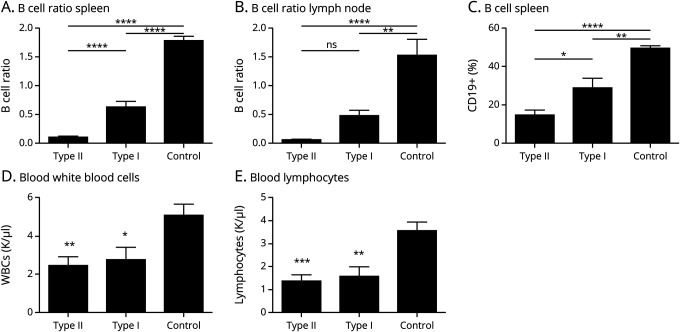
Anti-CD20 Depletes Peripheral B Cells in the DTH-TLS Model DTH-TLS lesions were established in huCD20 mice. Forty days after lesion induction, the mice were treated with either type I anti-CD20, type II anti-CD20, or a vehicle control. Twenty days after treatment, blood and organs were collected and analyzed. (A and C) Both type II and type I therapy caused a reduction in B cells in the spleen. Type II depleted splenic B cells better than type I. (B) The B-cell proportion in the lymph nodes was also reduced after both type I and II treatment. There was no difference between the 2 treatment antibodies. (D) The number of circulating white blood cells was reduced after anti-CD20 therapy. (E) Type I and II both reduced the number of blood lymphocytes compared with controls. Data are mean ± SEM *p < 0.05, **p < 0.01, ***p < 0.001, and ****p < 0.0001. Statistics: 1-way ANOVA with Tukey multiple comparisons test (type I n = 9, type II n = 11, control n = 13). ANOVA = analysis of variance; TLS = tertiary lymphoid structures; WBC, white blood cell.

Forty-two days after DTH-TLS lesion induction, mice were treated with either type I anti-CD20 (RTX), type II anti-CD20 (GA101), or control. After treatment, the effects of the therapies on B-cell depletion were examined. Both type II and type I reduced the area covered by B cells compared with controls in the spleen (type II: 0.1002 ± 0.019, type I: 0.6258 ± 0.100 vs control: 1.786 ± 0.0761 [*p* < 0.0001]) (figure 3A) and lymph nodes (type II: 0.0514 ± 0.0069, [*p* < 0.0001]; type I: 0.4724 ± 0.0906 [*p* = 0.0090] vs control: 1.525 ± 0.2810) (figure 3B). Type II depleted more splenic B cells than type I (*p* < 0.0001), although a difference was observed between the therapies on lymph node B-cell depletion, this was not statistically significant (*p* = 0.4602). Flow cytometry revealed a similar B-cell depletion pattern in the spleen; type II Ab depleted more B cells than type I Ab (*p* = 0.0156) (type II: 14.43 ± 2.549 *p* < 0.0001; type I: 28.68 ± 5.060, *p* = 0.0015 vs control: 49.42 ± 1.178) (figure 3C). Treatment with both anti-CD20 Abs further resulted in a decrease in the total white blood cell count in the blood (type II: 2.285 ± 0.446, type I: 2.648 ± 0.652 vs control: 4.908 ± 0.594 K/μL; *p* = 0.0052 for type II and *p* = 0.0310 for type I) (figure 3D). Assessment of the lymphocyte compartment showed that type II Ab reduced blood lymphocyte counts to 1.499 ± 0.280 (*p* = 0.0006) and type I to 1.480 ± 0.411 (*p* = 0.0015) compared with 3.358 ± 1.58 K/μL in vehicle-treated mice (figure 3E). The decrease in circulating lymphocytes and white blood cells was not different between the type I– and the type II Ab–treated mice (*p* = 0.9995 and *p* = 0.9142, respectively).

### Anti-CD20 Treatment Reduces Microglial and Astrocyte Activation in DTH-TLS Lesions

**Figure 4:**
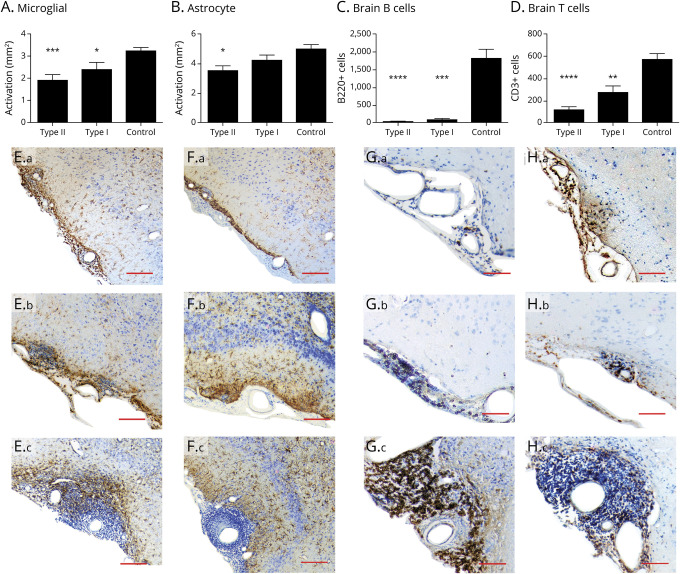
Anti-CD20 Therapy Reduces the Number of Lymphocytes and Glial Activation in DTH-TLS Lesions DTH-TLS lesions were established in huCD20 mice. Forty days after lesion induction, the mice were treated with either type I anti-CD20, type II anti-CD20, or a vehicle control for 15 days. (A) Immunohistochemistry showed a reduction in the area of microglial activation after both anti-CD20 treatments. (B) Only type II reduced the area of astrocyte activation. (C) Type I and type II both reduced the number of B cells in the brain. (D) Both anti-CD20 antibodies also lowered the number of T cells in the brain. (E) Photomicrographs show activated microglia in the lesions type II–treated animals (a), type I–treated animals (b), and control animals (c). (F) Representative photomicrographs show astrocytes activation in type II–treated animals (a), type I–treated animals (b), and control animals (c). (G) Representative photomicrographs of meningeal B cells in type II– (a), type I– (b), and vehicle-treated mice (c). (H) Representative photomicrographs of meningeal T cells in type II– (a), type I– (b), and vehicle-treated mice (c). Data represent mean ± SEM; *p < 0.05, **p < 0.01, ***p < 0.001, and ****p < 0.0001. Statistics: 1-way ANOVA with Tukey multiple comparisons test, n = 8 for type II, n = 6 for type I, and n = 11 for control. Scale bars represent 100 µm. ANOVA = analysis of variance; TLS = tertiary lymphoid structures.

To compare the effect of anti-CD20 on MS-like lesions, the area of microglial and astrocyte activation was determined. Anti-CD20 therapy reduced the area of activated microglia. Type II Ab reduced the area of activated microglia to 1.895 mm^2^ ± 0.249 mm^2^ (*p* = 0.0002) and type I Ab to 2.371 mm^2^ ± 0.341 mm^2^ (*p* = 0.0467) compared with 3.222 mm^2^ ± 0.153 mm^2^ in the control-treated mice (figure 4, A and E). No difference between the effects of the 2 antibodies was observed (*p* = 0.408).

Only type II Ab had an effect on the area of astrocyte activation. Type II reduced the area to 3.532 mm^2^ ± 0.341 mm^2^ (*p* = 0.012), whereas type I Ab reduced the area to 4.424 mm^2^ ± 0.358 mm^2^ (*p* = 0.370) compared with control (4.982 mm^2^ ± 0.315 mm^2^) (figure 4, B and F). In type II–treated mice, the astrocytes tended to cluster focally at the parenchymal-meningeal interface, forming a glial scar, whereas in type I– and control-treated mice, the astrocytes spread further out (figure 4F).

### Anti-CD20 Therapy Reduces the Number of B and T Cells in the Brain

Next, the effect of anti-CD20 antibodies was examined on brain B and T cells and the meningeal aggregates. Complete shrinkage of the TLS was observed (figure 4, C and G). Analysis showed that both anti-CD20 antibodies reduced the total number of B cells in the ipsilateral hemisphere (type II: 31.78 ± 9.260, *p* < 0.0001; type I: 82.60 ± 36.63, *p* < 0.001 vs control: 1,805 ± 265.6). No difference in brain B cell depletion was observed between the two therapies.

Anti-CD20 treatment lowered the number of T cells in the meninges and brain parenchyma (figure 4, D and H). Very few T cells were left in the meninges and around cuffed vessels in the brain parenchyma. Type II Ab lowered the number of T cells to 116.1 ± 26.71 (*p* < 0.0001), whereas type I Ab lowered the T-cell number to 270 ± 63.89 (*p* < 0.01) compared with vehicle-treated animals (567 ± 55.94). Type I and type II Abs did not have significantly different effects on reducing the number of brain T cells (*p* = 0.266) (figure 4D).

### Type II anti-CD20 Protects Neurons in the DTH-TLS Model

**Figure 5:**
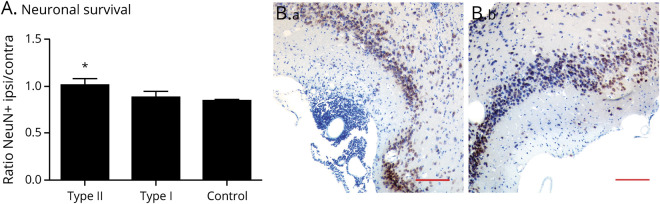
GA101 Protects Neurons in the DTH-TLS Model DTH-TLS lesions were established in huCD20 mice. Forty days after lesion induction, the mice were treated with either type I anti-CD20, type II anti-CD20, or a vehicle control. Neurons were immunolabeled (NeuN), and their numbers were counted in the piriform cortex in the ipsilateral and contralateral hemisphere. (A) Type II improves the ratio of surviving neurons in the ipsilateral piriform cortex. (B) Representative photomicrographs of neurons stained in the ipsilateral (a) and contralateral (b) piriform cortex of vehicle-treated animals. Data are presented as mean ± SEM. *p < 0.05. Statistics: 1-way ANOVA with Tukey multiple comparison test, n = 8 for type II, n = 6 for type I, and n = 11 for control. Scale bar represents 100 μm. TLS = tertiary lymphoid structures.

To determine the effect of anti-CD20 on neuronal survival in the DTH-TLS model, neuronal cell bodies were counted in the piriform cortex in the ipsilateral and contralateral hemisphere, and their ratio was calculated (figure 5). The ratio between ipsilateral and contralateral was 1.001 ± 0.070 for type II anti-CD20–, 0.886 ± 0.058 for type I–, and 0.850 ± 0.014 for vehicle-treated animals. Mice treated with type II had a higher ratio of ipsilateral vs contralateral neurons than control-treated mice (*p* = 0.0480). Lying between the groups, the effect of the type I anti-CD20 treatment on neuronal survival was not significantly different from that of the vehicle treatment group (*p* = 0.876), but nor was it different from the effect of the type II Ab (*p* = 0.249) (figure 5A). Furthermore, anti-CD20 therapy reduced myelin phagocytosis throughout the lesion; double-labeling for Mac-2 and MBP revealed that both type I– and type II anti-CD20–treated animals had fewer myelin-phagocytosing macrophages in the brain (86.17 ± 13.98, *p* = 0.04; 56.75 ± 8.394, *p* = 0.0003, respectively, compared with control 133.1 ± 2.64). Although there was a numerical difference between the effect of the 2 anti-CD20 Abs, there was no statistical difference (*p* = 0.081) (figure e-2, links.lww.com/NXI/A432).

## Discussion

Although progress has been made in the development of therapies for RMS, therapies are still lacking for patients with SPMS. The pathophysiology differs between SPMS and RMS. Moreover, in a subgroup of patients with SPMS, meningeal TLSs are present, which are associated with a more severe disease course.^4,7,8^ TLS is an understudied topic in MS, owing to the lack of imaging tools and tissue samples. To address this unmet need, this study described a new mouse model, the DTH-TLS model. The focal lesions mimic MS histologically in terms of microglial activation, astrogliosis, demyelination, and lymphocyte infiltration and are clinically silent (figure e-2, links.lww.com/NXI/A432). Furthermore, the model features structured and long-term aggregates of lymphocytes in the meningeal-brain parenchymal interface, representing TLS. A similar histologic picture can be achieved by targeting the striatum; however, no meningeal aggregates can be observed (figure e-3, links.lww.com/NXI/A433). This could be explained by the different level of vessel permeability between the meninges and the brain parenchyma or simply the available space for structures to expand.^19^ Injecting TB into the piriform cortex of nonimmunized mice causes local glial activation, but no lymphocyte infiltration, which is in line with previously reported experiments (figure e-3).^20^ Furthermore, we found that MOG immunization was necessary for the focal brain lesions to develop. Our model is based on the DTH (delayed-type hypersensitivity) model, which was the first focal model of MS-like bystander tissue destruction described in rats. The DTH model employs similar methods to those used in our model, and gives rise to T-cell mediated demyelinating brain lesions, in absence of any B cells. Our new paradigm, which makes use of MOG immunization, encouraged B-cell recruitment to the lesions, which is not a feature of the DTH lesions rats.^20^

Chronic active lesions in progressive MS and lesions with meningeal TLS are associated with increased numbers of activated microglia, especially around TLS.^6,7,21–24^ Such patterns of microglia were also present in DTH-TLS lesions. Astrogliosis was another prominent feature, and the area of activated astrocytes was found to map the same regions as activated microglia. Whether microglial activation gives rise to astrocyte activation or vice versa remains unclear. The presence of microglia has been shown to be essential for the development of EAE, but, as yet, the relative role of astrocytes in lesion formation has not been determined. Astrocytes are known to form glial scars in progressive MS lesions, a phenomenon represented in the DTH-TLS model in the late time points.^25,26^

B and T cells are important components of TLS.^4,8^ In the DTH-TLS model, we demonstrated that both cell types are present in the meninges from day 1 and that their numbers increase over time. From day 28 onward, the 2 cell types started to compartmentalize.^5,27^ Moreover, we showed presence of plasma cells and in situ proliferation of B cells in the aggregates, which are other aspects of TLS.^6,8^ It should be noted that meningeal inflammation is not MS specific, and high numbers of B cells are also present in case of B-cell lymphoma or TB meningitis.^7,28–30^ We further showed that the development of TLS was associated with ongoing demyelination. Patients with SPMS with meningeal TLS showed more severe demyelination, which corroborates what we observed in our model.^6,8,22,31^

As final part of the model characterization, we investigated the status of the BBB. In progressive MS, chronic active lesions often evolve behind an intact BBB, that is, no gadolinium enhancement is detectable on MRI.^21,32–34^ Absence of gadolinium enhancement in MRI was also observed in the DTH-TLS lesions at day 100, which indicates no major BBB breakdown at this time point.

The utility of the model to test potential therapy for progressive disease was explored using 2 types of anti-CD20: RTX (type I) and GA101 (type II). GA101 was found to deplete peripheral B cells more effectively than RTX, which is in agreement with previous studies.^12,13,16^ Whether enhanced peripheral B-cell depletion is required for improved therapeutic efficacy in MS is unclear, but incomplete B-cell depletion is associated with increased antidrug antibodies and lower safety profile.^35^ In the DTH-TLS model, both anti-huCD20 antibodies reduced the extent of microglial activation, which has been previously reported in rat models of MS using rodent anti-CD20 antibodies.^36^ Of interest, only the type II Ab reduced astrocyte activation. This effect is difficult to explain in relation to B-cell or T-cell depletion because there was no significant difference in the magnitude of the reduction between the treatment groups. It is a limitation of our study that it lacks pharmacokinetics data for the brain bioavailability of the 2 antibodies, and some of the observed difference may be a consequence of altered patterns of distribution rather than mode of action. In numerical terms, the level of depletion of both B cells and T cells was greater for the type II in the brain. Future studies that compare anti-CD20 therapies at different doses may elucidate the impact of anti-CD20 therapy on different outcomes. However, the mode of cell death induced by the 2 antibodies might play a role. RTX relocates CD20 to lipid rafts and induces significant CDC and Ab‐dependent cellular cytotoxicity. GA101 has reduced CDC activity, but, as a consequence of alternative binding to the CD20 molecule, it induces greater direct cell death. Furthermore, GA101 is known to increase phagocytosis, which might lead to decreased cytokine production that might affect astrocyte function. Anti-CD20 antibodies have been shown to deplete B cells in both the parenchyma and in the CSF of patients.^16,37,38^ Surprisingly, the depletion of the CD20^+^ population was able to significantly reduce the total size of the meningeal aggregates and affected all cell types. In the spleen, it is of note that although anti-CD20 eliminates the B cells, the T-cell population remains largely intact.^36^ Thus, in the brain at least, the continued structural organization of the TLS-like structures seems to rely on the presence of B cells, which underlines the difference between the organization of secondary and tertiary lymphoid organs.

We further showed that type II anti-CD20 therapy protected from neuronal death in this model, whereas treatment with RTX did not confer any significant protection compared with control-treated animals. The presence of TLS in MS correlates with increased cortical atrophy and neuronal loss, and any ability to reduce bystander cell loss is likely to have an impact on clinical progression.^7^ The cause of the increased neuronal loss adjacent to TLS in MS brains remains unknown, but models, such as the one described here, provide an important tool to better understand this process. The decreased number of myelin-positive macrophages throughout the lesion following anti-CD20 therapy indicates that there is a reduction in the amount of structural damage associated with the lesion, which might contribute to the preservation of neurons. Together, the results reveal that anti-CD20 treatment reduces neuroinflammation, eliminates TLS, and augments neuronal survival in the DTH-TLS model in mice, but the increased efficacy of the type II anti-CD20 Ab suggests that its use in the clinic might be more beneficial for progression disease than the type I anti-CD20 antibodies that have been used to date, which have only had a modest effect on progression.

In conclusion, we have demonstrated the treatment potential of anti-CD20 antibodies in a new focal model of progressive MS. The model resembles SPMS histologically, including meningeal lymphocytic aggregates and an intact BBB. Here, we compared 2 anti-CD20 therapies and their effects on the TLS and inflammation in this model. We showed that the type II anti-CD20 caused a more complete peripheral B-cell depletion than type I, which is associated with a larger reduction of microglial and astrocyte activation and the size of the meningeal aggregates. Finally, we showed that the type II Ab prevents neuronal death in this model. Taken together, these results show that GA101 is likely to be a promising treatment for progressive MS.

## Glossary

Ab: antibody
BBB: blood-brain barrier
CDC: complement-dependent cytotoxicity
MBP: myelin
PFA: paraformaldehyde
RMS: relapsing MS
RTX: rituximab
SPMS: secondary progressive MS
TB: tuberculosis
TLS: tertiary lymphoid-like structure
